# Supplementary material for: Development of a Matrix Solid-Phase Dispersion Extraction Combined with UPLC/Q-TOF-MS for Determination of Phenolics and Terpenoids from the Euphorbia fischeriana
Source: Molecules. 2017 Sep 11;22(9):1524. doi: 10.3390/molecules22091524 (PMC6151458; doi:10.3390/molecules22091524)
Supplement: Supplementary File 1 [file molecules-22-01524-s001.pdf]

molecules

Development of a matrix solid-phase dispersion extraction combined with UPLC/Q-TOF-MS for determination of phenolics and terpenoids from the *Euphorbia fischeriana*

Wenjing Li, Yu Lin, Yuchun Wang, Bo Hong*

College of Pharmacy, Qiqihar Medical University, Heilongjiang 161006, China; lwj022325@163.com(W.L.); linyu197312@126.com(Y.L.); yuchunwang1978@gmail.com(Y.W.);
Correspondence: bohong200630174@163.com(B.H.); Tel.: +86-452-2663159(B.H.)

Abstract: A method based on a simplified extraction by matrix solid phase dispersion (MSPD) followed by ultra-performance liquid chromatography coupled with the quadrupole time-of-flight tandem mass spectrometry (UPLC/Q-TOF-MS) determination is validated for analysis of two phenolics and three terpenoids in *Euphorbia fischeriana*. The optimized experimental parameters of MSPD including dispersing sorbent (silica gel), ratio of sample to dispersing sorbent (1:2), elution solvent (water-ethanol: 30-70) and volume of the elution solvent (10 mL) were examined and set down. The highest extraction yields of chromatogram information and the five compounds were obtained under the optimized conditions. A total of 25 constituents have been identified and five components have been quantified from *Euphorbia fischeriana*. A linear relationship ($r^2 \geq 0.9964$) between the concentrations and the peak areas of the mixed standard substances were revealed. The average recovery were between 92.4% and 103.2% with RSD values were less than 3.45% (n=5). The extraction yields of two phenolics and three terpenoids obtained by the MSPD were higer than those of traditional reflux and sonication extraction with reduced requirement on sample, solvent and time. In addition, the optimized method will be applied for analyzing terpenoids in other Chinese herbal medicine samples.

Keywords: *Euphorbia fischeriana*; phenolics; terpenoids; Matrix solid-phase dispersion extraction; UPLC/Q-TOF-MS;

1. Introduction

People in China and other Asian countries have used Traditional Chinese Medicine (TCM) to treat various diseases for centuries. *Euphorbia fischeriana* also known as "Langdudaji" in China, the root of *E. fischeriana* Steud., is one of the most famous TCM herbs. It has been used in many TCM formulations for thousands of years. It has been used for the treatment of edema, phlegm accumulation, inflammation, ascites and cancer in clinical practice for many years and show great efficacy [1-3]. Modern medical research showed that the extracts of *E. fischeriana* were found to inhibit the growth of Lewis lung carcinoma and ascetic hepatoma in mice [4]. Hot AcOEt extracts and cold Et_2O extracts of *E. fischeriana* showed most effective inhibition rates on tuberculosis bacillus in vitro [5]. The crude extracts of *E. fischeriana* can increase survival rate of the mice inoculated with L_{615} leukemia. Xinchao Liu obtained jolkinolide A, jolkinolide B and 17-hydroxyjolkinolide B from ethanol extracts of *Euphorbia fischeriana* exhibited nematicidal activity [6]. Previous studies of this plant have shown that it mainly contains diterpenoids [7-8], triterpenoids [9] and steroids [10]. Terpenoids which have a isoprene or isopentane type skeleton are considered the major constituents and the main bioactive ingredients in the *E. fischeriana*. Up to now, more than 40 of these terpenoids have been isolated from various parts of *E. fischeriana*[11]. Usually Scopoletin, 2, 4-dihydroxy-6-methoxy-3- methylacetophenone, 17-Hydroxyjolkinolide B,

Jolkinolide B and Jolkinolide A are the highest abundance among these founded terpenoids. The main terpenoids extract have been demonstrated to possess similar pharmacological bioactivity, including strong antitumor activity against several tumor lines such as human prostate, hepatic carcinoma, and Leukemia cancer [12-13], antituberculosis effect [14], antibacterial effect [15]. Scopoletin possess enhancing effects on lymphocyte mitogen responsiveness, it could be a potential antitumoral compound to used for cancer treatment [16]; 2,4-dihydroxy-6-methoxy-3-methylacetophenone has inhibitive activity against mycobacterium tuberculosis [17]; 17-Hydroxyjolkinolide B can inhibit growth and induce apoptosis of tumor cells, which is a promising anticancer drug candidate as a potent signal transducer and activator of transcription signaling inhibitor [18]; Jolkinolide B has the anti-proliferation effect on human chronic myeloid leukemia cells [19-20]; Jolkinolide A has a significant inhibition activity of the growth of cells of S-180 and ehrlich's ascites carcinoma [21].

Due to the wide range of biological activities, the use of *E. fischeriana* has increased vigorously and gained popularity. However, the content and distribution of terpenoids in *E. fischeriana* are affected by different plant origins and harvest seasons. Therefore, a simple and rapid method to extract and determine major terpenoids, especially the two phenolics and three terpenoids in *E. fischeriana* is extremely desirable to quality control of *E. fischeriana*. The herb and the five compounds structures are shown in Fig.1. Up to now, high performance liquid chromatography (HPLC) is the most common methods to analyze the major terpenoids in *E. fischeriana* [22-23]. Mass spectrometry hyphenated techniques to liquid chromatography (LC-MS/MS) [24] and high performance liquid chromatography combined with evaporative light scattering detection (HPLC-ELSD) [25] are also applied to analyze the major terpenoids in *E. fischeriana* as well. During these analytical process, the sample extraction methods including heat reflux, soxhlet or sonication were usually used to extract terpenoids from *E. fischeriana*. However, these conventional methods were high solvent consumption, time consuming, requiring additional clean-up, filtration in addition to concentration steps. Until now there is no effective standardized extraction method for analyzing terpenoids in *E. fischeriana*. Therefore, an alternatively simple and effective extraction method is of great necessary in the recent years.

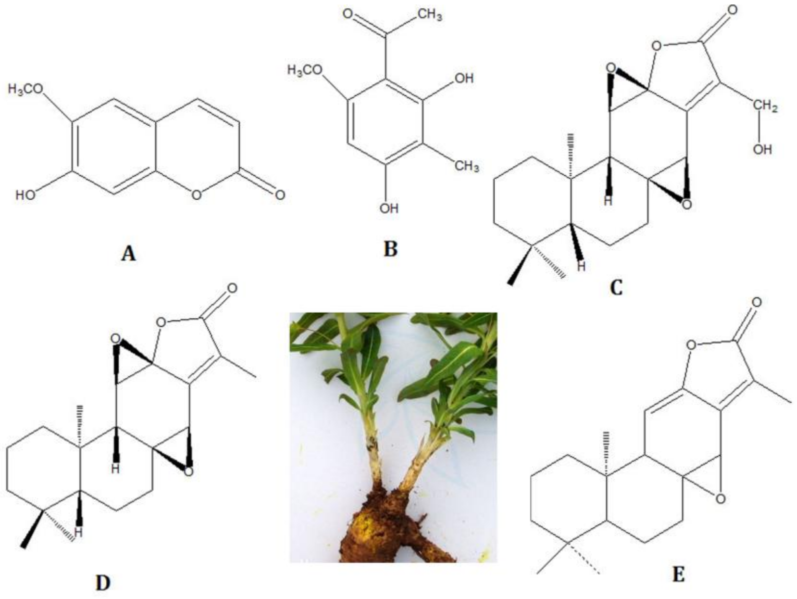

Fig.1. The herb and chemical structures of the five reference substances. Scopoletin (A), 2,4-dihydroxy-6-methoxy-3-methylacetophenone (B), 17-Hydroxyjolkinolide B (C), Jolkinolide B (D), Jolkinolide A (E).

Barker firstly introduced matrix solid phase dispersion (MSPD) technology in 1989 for the extraction of drug residues from animal tissue [26]. Since then, the technique has induced

considerable interests because of the unique properties of MSPD providing the simple, low-cost and convenient benefits. As one of the most promising techniques, MSPD has been successfully applied to solve many difficult analytical problems. Recnetly, MSPD has been applied more and more frequently as a potential and effective alternative to conventional extraction methods in the extraction of active ingredients from medicinal plants [27-28]. However, there is no report on MSPD as a extraction method for the simultaneous extraction of active compounds mainly the terpenoids from *E. fischeriana*. in the literature to our best knowledge.

In this study, to achieve the maximum extraction yield, MSPD as alternative sample preparation method followed by UPLC/Q-TOF-MS separation was applied to extract and determine the main two phenolics and three terpenoids and other components in *E. fischeriana*. The effects of MSPD extraction for terpenoids were evaluated and optimized by various operating parameters, including dispersing sorbent, elution solvent and volume, the ratio of dispersing sorbent to sample. Then a validation for HPLC method, including linearity, precision, accuracy and so on were evaluated. MSPD-UPLC/Q-TOF-MS, as a powerful hyphenated technique, was used for characterization of the main components in *E. fischeriana*. In addition, we compare the extraction yield obtained by the MSPD developed with those obtained by conventional extraction methods. It was expected that this research would be helpful for control the quality and make sure clinical therapeutic efficacy of *E. fischeriana*.

2. Results and Discussion

2.1. Optimization of MSPD extraction procedure

In order to achieve the highest extraction yields for the two phenolics and three terpenoids from *E. fischeriana*, the most suitable extraction parameters including type of dispersing sorbent, volume of the eluting solvent and the ratio of dispersing sorbent to sample were evaluated through determination of the extraction yield and the purity of the final extract.

2.1.1. Selection of dispersing sorbent

Four kinds of frequent dispersing sorbents, including Silica gel, florisil, neutral alumina, C_{18}-bonded silica were tested in this step. The results of extraction yields of terpenoids from *E. Fischeriana* obtained with the four different dispersing sorbents were shown in Table 1. As can be seen, when we used the silica gel, the extraction yields which is calculated by the ratio of extracted compound to medicinal material of the two phenolics and three terpenoids were a little higher than the extraction yields with the other three sorbents. Therefore, silica gel was the dispersing sorbent selected for MSPD because of the best extraction yields for the two phenolics and three terpenoids and the relatively low cost.

Table 1. Extraction yields (%) obtained using different dispersion adsorbents(silica gel, florisil, neutral alumina, C_{18}-bonded silica).

Dispersion adsorbents	Scopoletin (%)	2,4-dihydroxy-6-methoxy-3-methylacetophenone(%)	17-Hydroxyjolkinolide B(%)	Jolkinolide B (%)	Jolkinolide A (%)
Silica gel	0.0042	0.0346	0.0964	0.1089	0.0279
florisil	0.0034	0.0297	0.0678*	0.0822	0.0254
neutral alumina	0.0023*	0.0247*	0.0496**	0.0466*	0.0198
C_{18}-bonded silica	0.0038	0.0337	0.072	0.0923	0.0268

Note: To compare with Silica gel group: *P<0.05, **P<0.01

2.1.2. Ratio of dispersing sorbent to sample

A best ratio of dispersing sorbent to sample could make sure the sample fully contact with the

dispersing sorbent. Therefore, four different mass ratios of sample to silica gel ranging from 1:1 to 1:4 were evaluated. The results were shown in Fig.2. It indicated that the extraction yields increase with the increase of mass ratios of sample to silica gel less than 1:2. Further increasing the mass ratio to 1:3 or 1:4 resulted in no significant increase even reduction of extraction yields of the two phenolics and three terpenoids. Thus, the optimized mass ratio was selected at 1:2 in this work.

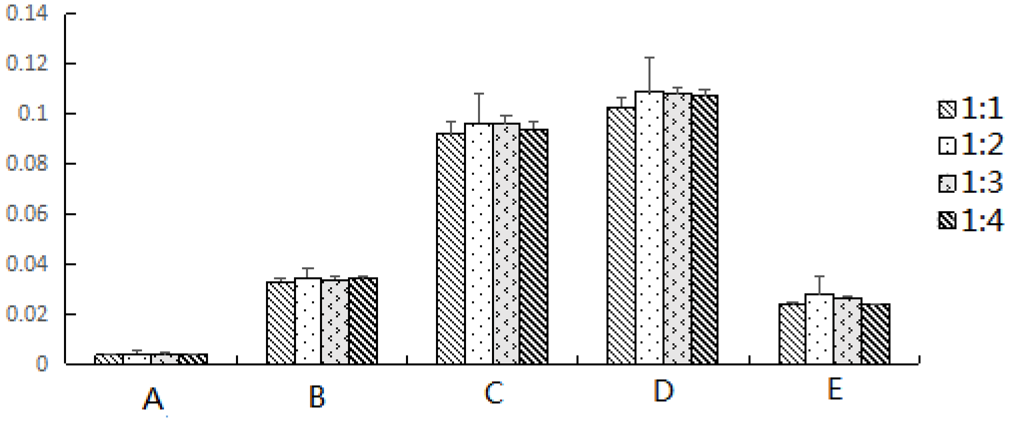

Fig.2. The effect of the ratio of sample to adsorbent on extraction yields (%) of Scopoletin (A), 2,4-dihydroxy-6-methoxy-3-methylacetophenone (B), 17-Hydroxyjolkinolide B (C), Jolkinolide B (D), Jolkinolide A (E) from *E. Fischeriana*.

2.1.3. Effect of elution solvents

The nature of the elution solvent is also an important factor in the MSPD procedure. The elution solvent can not only separate the chemical profile just like the mobile phase, but also dissolve the target compounds from sorbents. We must make sure that the target compounds were selectively desorbed while the other components were retained in the column. Because the terpenoids is soluble in ethanol solvent, therefore, four solvents with different polarity including water-thanol (20:80), water-ethanol (30:70), water-ethanol (50:50) and pure ethanol were evaluated to select the best solvent for extraction of the terpenoids from *E. Fischeriana.* The results of these experiments were presented in Fig.3. As we can seen form the results, the yields of the five target compounds were highest using the elution solvent of water-ethanol (30:70). However, the pure ethanol shew the worst performance.

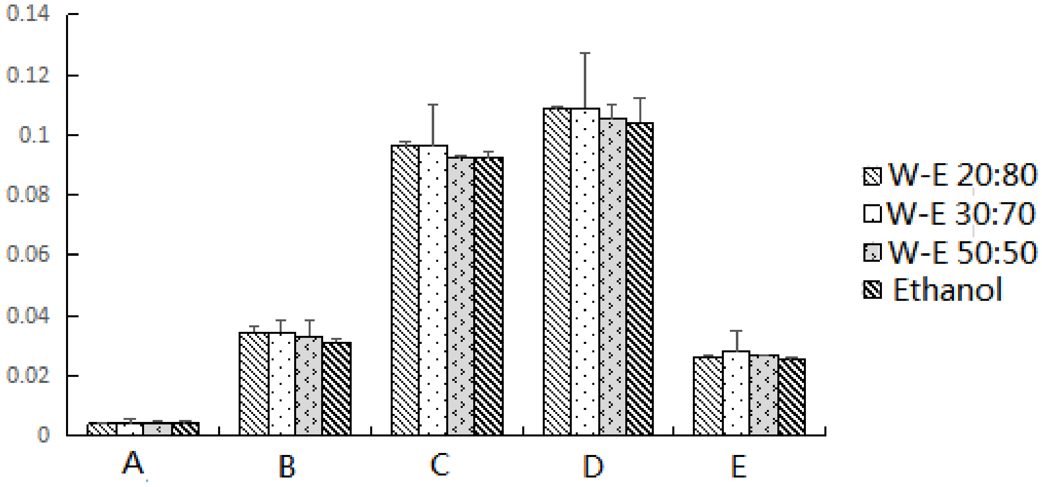

Fig.3. The effect of elution solvents on the extraction yields (%) of Scopoletin (A), 2,4-dihydroxy-6-methoxy-3-methylacetophenone (B), 17-Hydroxyjolkinolide B (C), Jolkinolide B (D), Jolkinolide A (E) from *E. Fischeriana*.

2.2. Optimization of UPLC conditions

To obtain desirable UPLC chromatograms, the procedure of sample separation was optimized in selecting the factors of extraction method, separation solvent and so on. Acetonitrile-water possessed better resolution and peak shape than methanol-water system. It was also found that good signal intensity, resolution and peak shape were achieved when 0.1% (*v/v*) formic acid was added to aqueous solution. To sum up, we determined the different kinds of compounds in *Euphorbia fischeriana* with mobile phase consisted of A (acetonitrile) and B (0.1% v/v aqueous formic acid). In order to screen and separate the components completely, a high-gradient slope was used. The UV detection wavelength was set at 210 nm, at which most components can be detected sensitively.

2.3. Procedure for identification of the components in Euphorbia fischeriana

Both the positive and negative ion modes were tested to characterize the chemical composition of *Euphorbia fischeriana*. Most compounds showed much cleaner mass spectral background and higher sensitivity in the positive mode than in the negative mode. The representative positive UPLC/Q-TOF-MS total ion chromatogram of *Euphorbia fischeriana* is presented in Fig.4. TOF-MS mode was used for further confirmation of the identity of the detected compounds in *Euphorbia fischeriana*, which furnished accurate molecular mass ions, used to obtain elemental compositions. The identity of known compounds in the herbal extract was confirmed by co-chromatography and comparing with reference standards (compounds 8, 18, 23, 24, 29) according to the retention time and molecular ions. A total of 29 compounds were characterized, 25 of which were identified by comparing the mass spectra and retention times with those of reference standards. Screening, identification and further confirmation of the components in *Euphorbia fischeriana* are shown in Table 2. A narrow window used for the extract ion chromatogram (EIC) leads to a more selective identification for the analytical compounds and reduces matrix interference (Fig.5). Due to absence of reference compounds, the compounds corresponding to the other 20 peaks were tentatively identified by MS determination the m/z value of $[M+H]^+$, $[M+Na]^+$ and $[M+K]^+$ ion. Compounnds 4, 5, 13, 15, 16, 19 and 21 gave protonated molecular ion $[M+H]^+$, compounds 6, 7, 9 and 22 gave molecular ion $[M+Na]^+$, compounds 10, 11, 12 and 27 gave molecular ion $[M+K]^+$, compounds 14, 20, 25, 26 and 28 gave molecular ion $[M+H]^+$ and $[M+Na]^+$.

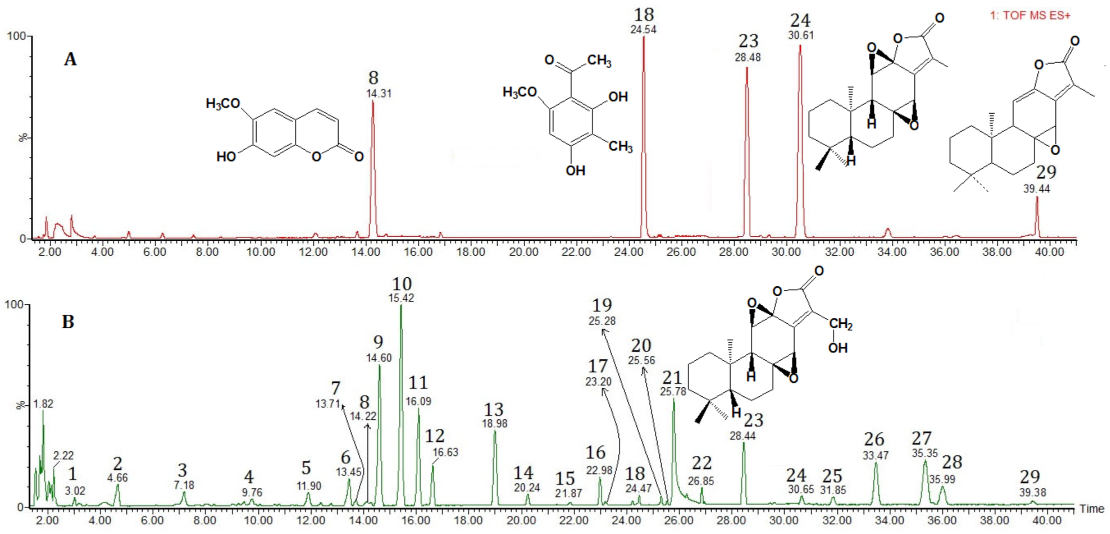

Fig.4. Representative UPLC-Q-TOF-MS chromatograms of: (A) TIC of reference stock solution (8. Scopoletin; 18. 2,4-dihydroxy-6-methoxy-3-methylacetophen one; 23. 17-Hydroxyjolkinolide B; 24. Jolkinolide B; 29. Jolkinolide A); (B) TIC of extract sample obtained from *Euphorbia fischeriana* in positive-ion mode.

Table 2. Components of *Euphorbia fischeriana* identified by UPLC-Q-TOF-MS in positive-ion mode

Peak No.	t_R (min)	Elemental composition	Assigned identity	Theoretical mass (m/z)	Experimental mass (m/z)	Error (m m/z units)
1	3.02				132.1028	
2	4.66				166.0867	
3	7.18				205.0510	
4	9.76	$C_{23}H_{38}O_3$	3β,16β,17-trihydroxy-ent-kaurane 16,17-acetonide [30]	363.2899 $[M+H]^+$	363.2848	-5.1
5	11.90	$C_{28}H_{40}O_{12}$	Fischerosides C [31]	569.2598 $[M+H]^+$	569.2621	-2.3
6	13.45	$C_{30}H_{24}O_{10}$	Chamechromone [32]	567.4953 $[M+Na]^+$	567.4905	-4.8
7	13.71	$C_{20}H_{34}O_3$	Ent-atisane-3β,16α,17-triol [33]	345.2406 $[M+Na]^+$	345.2386	-2.0
8	14.22	$C_{10}H_8O_4$	Scopoletin [34]	193.0423 $[M+H]^+$ 215.0320 $[M+Na]^+$	193.0494, 215.0305	7.1, -1.5
9	14.60	$C_{20}H_{30}O_3$	Kauranoic acid [35]	341.2093 $[M+Na]^+$	341.2021	-7.2
10	15.42	$C_{16}H_{22}O_9$	2,4-dihydroxy-6-methoxy-3-methylacetophenone-4-O-β-D- glucopyranoside [36]	397.4388 $[M+K]^+$	397.4324	-6.4
11	16.09	$C_{29}H_{50}O$	β-sitosterol [37]	453.3499 $[M+K]^+$	453.3437	-6.2
12	16.63	$C_{26}H_{36}O_9$	Fischeriana B [38]	531.6569 $[M+K]^+$	531.6565	-0.4
13	18.89	$C_{28}H_{40}O_{11}$	Fischerosides A [30]	553.2649 $[M+H]^+$	553.2646	-0.3
14	20.24	$C_{35}H_{44}O_{15}$	Fischerosides B [30]	705.2758 $[M+H]^+$, 727.2578 $[M+Na]^+$	705.2756, 727.2574	-0.2, -0.4
15	21.87	$C_9H_{10}O_4$	2,4-dihydroxy-6-methoxy-acetophenone [39]	183.0657 $[M+H]^+$	183.0639	-1.8
16	22.98	$C_{22}H_{28}O_5$	17-acetoxyjolknolide A [11]	373.2015 $[M+H]^+$	373.2004	-1.1
17	23.20				353.2279	
18	24.49	$C_{10}H_{12}O_4$	2,4-Dihydroxy-6-methoxy-3-methylacetophenone [40]	197.0814 $[M+H]^+$	197.0806	-0.8
19	25.28	$C_{20}H_{32}O_3$	Ent-kaurane-3-oxo-16α, 17-diol [41]	321.2430 $[M+H]^+$	321.2401	-2.9
20	25.56	$C_{20}H_{28}O_4$	Ebracteolatanolide A [42]	333.2066 $[M+H]^+$, 355.1885 $[M+Na]^+$	333.2062, 355.1890	-0.4, 0.5
21	25.78	$C_{21}H_{34}O_3$	17-dihydroxy-ent-atisan-19-oic acid methyl ester [43]	335.2586 $[M+H]^+$	335.2592	0.6
22	26.85	$C_{20}H_{28}O_5$	Langduin A [13]	371.1834 $[M+Na]^+$	371.1821	-1.3
23	28.44	$C_{20}H_{26}O_5$	17-hydroxyjolkinolide B [44]	347.1859 $[M+H]^+$, 369.1678 $[M+Na]^+$	347.1845, 369.1684	-1.4, 0.6

24	30.65	$C_{20}H_{26}O_4$	Jolkinolide B [44]	331.1909 $[M+H]^+$, 353.1729 $[M+Na]^+$	331.1913, 353.1716	0.4, -1.3
25	31.85	$C_{20}H_{28}O_3$	Ent-11β-hydroxyabieta-8 (14), 13(15)-dien-16-12β-olide [11]	317.2117 $[M+H]^+$, 339.1936 $[M+Na]^+$	317.2112, 339.1930	-0.5, -0.6
26	33.48	$C_{20}H_{26}O_4$	17-hydroxyjolkinolide A [44]	331.1909 $[M+H]^+$, 353.1729 $[M+Na]^+$	331.1906, 353.1723	-0.3, -0.6
27	35.35	$C_{20}H_{26}O_4$	Fischeriana A [38]	369.1468 $[M+K]^+$	369.1432	-3.6
28	35.99	$C_{16}H_{22}O_4$	Dibutyl phthalate [45]	279.1596 $[M+H]^+$, 301.1416 $[M+Na]^+$	279.1604, 301.1421	-0.8, 0.5
29	39.38	$C_{20}H_{26}O_3$	Jolkinolide A [44]	315.1960 $[M+H]^+$	315.1946	-1.4

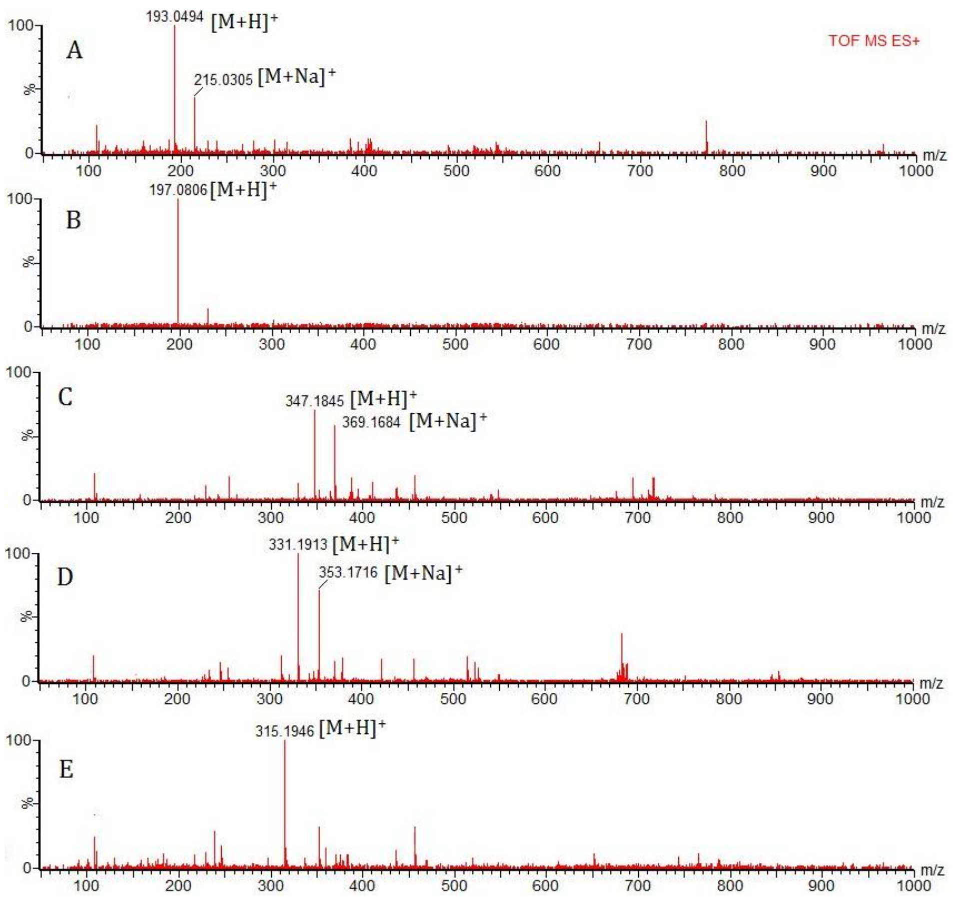

Fig.5. EIC of five compounds from *Euphorbia fischeriana* for quantification. A: Scopoletin with $[M+H]^+$ and $[M+Na]^+$ peak; B: 2, 4-dihydroxy-6-methoxy-3-methylacetophenone with $[M+H]^+$ peak; C: 17-Hydro xyjolkinolide B with $[M+H]^+$ and $[M+Na]^+$ peaks; D: Jolkinolide B with $[M+H]^+$ and $[M+Na]^+$ peaks; E: Jolkinolide A with $[M+H]^+$ peak.

2.4. Quantification method validation

Good linear calibration curves were obtained with 5 tested reference standards (r^2>0.9964) in the concentration range. The values of LOD and LOQ were in the range from 18.94 to 94.70 ng/mL and from 62.50 to 312.50 ng/mL respectively. The results show that the instrument has the desirable sensitivity to meet the quantitative requirements (Table 3).

Table 3. Calibration curves of Scopoletin, 2,4-dihydroxy-6-methoxy-3-methylacetophenone, 17-Hydroxyjolkinolide B, Jolkinolide B, Jolkinolide A in *Euphorbia fischeriana for quantification*.

**Compounds**	**Regression equation**	**Confidence intervals**	R^2	**Linear range (μg/mL)**	**LOD (ng/mL)**	**LOQ (ng/mL)**
Scopoletin	*Y*=239.93*X*-7.6326	223.31-256.56	0.9964	0.625-50	23.67	78.12
2,4-dihydroxy-6-methoxy -3-methylacetophenone	*Y*=27.32*X*+0.1943	25.85-28.80	0.9978	2.5-200	75.76	250.00
17-Hydroxyjolkinolide B	*Y*=127.9*X*+19.289	120.18-135.61	0.9973	1.25-100	47.35	156.25
Jolkinolide B	*Y*=29.571*X*-3.6311	27.65-31.49	0.9968	2.5-200	94.70	312.50
Jolkinolide A	*Y*=45.632*X*+6.3342	44.03-47.24	0.9991	0.625-50	18.94	62.50

To ensure correct quantification, precision of the proposed method was assessed by the relative standard deviation (RSD) values obtained from intra-day (within 1 day) and inter-day (3 consecutive days) precision, which were all less than 2.23%. The stability results showed that the sample solution was found to be stable within 48 h (RSD<3.09%). Validation studies of this method proved that this assay has good repeatability with a RSD less than 4.02% (*n*=5) for the 5 analytes (Table 4). The recovery for these markers ranged from 92.4 to 103.2%, with RSD ranging from 1.32 to 3.45%. Thus, this analytical procedure is accurate and sufficiently sensitive for the simultaneous quantification of the 5 tested reference standards in *Euphorbia fischeriana.*

Table 4 Precision, repeatability, and stability of Scopoletin, 2,4-dihydroxy-6-methoxy-3-methylacetophenone, 17-Hydroxyjolkinolide B, Jolkinolide B, Jolkinolide A in *Euphorbia fischeriana* expressed with RSD (%)

Compound	Precision RSD (%)			Repeatability (*n*=6)		Stability (48 h, *n*=3)	
	Concentration (μg/mL)	Intraday (*n*=6)	Interday (*n*=3)	Content (%)	RSD (%)	Content (%)	RSD (%)
Scopoletin	6.25	1.12	2.23	0.0032	2.29	0.0029	2.05
2,4-dihydroxy-6-methoxy -3-methylacetophenone	25	0.93	1.21	0.0243	2.98	0.0241	2.57
17-Hydroxyjolkinolide B	12.5	1.29	1.43	0.0585	4.02	0.0581	2.98
Jolkinolide B	25	0.31	1.39	0.0594	3.21	0.0591	2.12
Jolkinolide A	6.25	1.09	1.87	0.0112	3.99	0.0114	3.09

2.5. Quantification of 5 compounds in the Euphorbia fischeriana

The established analytical method in this paper was successfully applied to simultaneously determine 5 active compounds in 5 different samples of *Euphorbia fischeriana* obtained from different cultivated areas. All of the contents are summarized in Table 5. The results suggest that there is a difference in the contents of the five marker compounds among the raw herbal materials, which may result from the difference in the place of origin. Among the samples, the concentration range of Scopoletin was 0.0028%-0.0043%; 2,4-dihydroxy-6-methoxy-3-methylacetophenone was 0.0285%-0.0453%; 17-Hydroxyjolkinolide B was 0.0524%-0.0943%; Jolkinolide B was

0.0454%-0.1045%; Jolkinolide A was 0.0112%-0.0284%. The highest concentration of Scopoletin was found in Harbin sample; the highest concentrations of 2,4-dihydroxy-6-methoxy-3-methylacetophenone and Jolkinolide A were found in Mudanjiang; the highest concentrations of 17-Hydroxyjolkinolide B and Jolkinolide B were found in Qiqihar. The results showed that Heilongjiang province as the genuine regional place of *Euphorbia fischeriana* herb has a higher content of active compounds compared with those of other places because of the growth weather condition, harvest time and storage.

Table 5 Contents of Scopoletin, 2,4-dihydroxy-6-methoxy-3-methylacetophenone, 17-Hydroxyjolkinolide B, Jolkinolide B, Jolkinolide A of *Euphorbia fischeriana* samples produced in Qiqihar, Harbin, Mudanjiang, Baoding, Changchun (*n*=3).

No.	Origins	Average Content (%) (*n*=3)				
		Scopoletin	2,4-dihydroxy-6-methoxy-3-methylacetophenone	17-Hydroxyjolkinolide B	Jolkinolide B	Jolkinolide A
1	Qiqihar	0.0032	0.0343	0.0943	0.1045	0.0112
2	Harbin	0.0043	0.0285	0.0885	0.0594	0.0205
3	Mudanjiang	0.0031	0.0453	0.0534	0.0454	0.0284
4	Baoding	0.0028	0.0293	0.0524	0.0506	0.0124
5	Changchun	0.0038	0.0405	0.0875	0.0498	0.0213

2.6. Comparison of MSPD, ultrasonic and reflux extraction

In order to evaluate the performances of optimized MSPD, the comparison among MSPD, ultrasonic and reflux extraction was made. The results of extraction yield are shown in Table 6. From the comparison results, it can be seen that there is an apparent poorer yield for ultrasonic extraction comparing with MSPD and reflux method. When reflux extraction was applied, much more sample, time and solvent were consumed comparing with MSPD. More important, the extraction condition was mild and the heating was not required during the MSPD procedure, thus the possible loss and degradation of the compounds could be avoided.

Table 6 Comparison the extraction yields of Scopoletin, 2,4-dihydroxy-6-methoxy-3-methylacetophenone, 17-Hydroxyjolkinolide B, Jolkinolide B, Jolkinolide A in *Euphorbia fischeriana* by MSPD, ultrasonic and reflux extraction methods

**Extraction yield (%)**	**MSPD**	**Ultrasonic**	**Reflux**
Scopoletin	0.0043	0.0039	0.0042
2, 4-dihydroxy-6-methoxy-3-methylacetophenone	0.0343	0.0327	0.0324
17-Hydroxyjolkinolide B	0.0971	0.0937	0.0963
Jolkinolide B	0.1056	0.0973*	0.1051
Jolkinolide A	0.0283	0.0264	0.0279

Note: Ultrasonic and Reflux groups compare with Silica gel group separately: **P*<0.05

3. Materials and methods

3.1. Chemicals and reagents

All chemicals and reagents used were of the highest grade available. Scopoletin, 2,4-dihydroxy-6-methoxy-3-methylacetophenone, Jolkinolide B were purchased from the National Institute for the Pharmaceutical and Biological Products (Beijing, China). The standards of 17-hydroxyjolkinolide B and Jolkinolide A were available from Qiqihar Medical University. The purity of all standards was above 98.0%. Formic acid was purchased from Kangkede Science and Technology Co., Ltd. (Tianjin, China). Methanol and acetonitrile (HPLC grade) were purchased from Dima technology Inc. (Richmond, VA, USA). Analytical grade methanol and ethanol were purchased from Tianjin Fuchen Chemical Factory (Tianjin, China). Ultra-pure water (18.2 MΩ) was

prepared with a PALL Purelab plus water purification system (Ann Arbor, MI, USA). Silica gel (200-300 mesh), Florisil (100-200 mesh) and neutral alumina (200 mesh) were obtained from Qingdao Haiyang Chemical Subsidiary Factory (Qingdao, China). C_{18}-bonded silica (200-300 mesh) was obtained from National Institute for the control of Pharmaceutical and Biological Products (NICPBP, Beijing, Chia). All solvents were filtered through a 0.22 μm membrane and were then degassed by sonication in an ultrasonic bath before use.

3.2. Plant materials

The *Euphorbia fischeriana* herbs were collected in Qiqihar, Harbin, Mudanjiang (Heilongjiang Province, China), Changchun (Jilin Province, China) and Baoding (Hebei Province, China) and verified as the genuine medicinal herbs by professor Lina Guo of Qiqihar Medical University. The voucher specimens are kept in the reference library for the medicinal herbs in Qiqihar Medical University.

3.3. Standard solution

The individual standard stock solutions (1 mg/mL) of Scopoletin (A), 2, 4-dihydroxy-6-methoxy-3-methylacetophenone (B), 17-Hydroxyjolkinolide B (C), Jolkinolide B (D), Jolkinolide A (E) were prepared by dissolving accurate amounts of pure standards in methonal. Mixed stock solution at a final concentration ranging from 50 to 200 μg/mL was dissolved in methanol. Series of working standard solutions were obtained by further dilution from the mixed stock solutions with methanol to prepare calibration curve. All solutions were stored at 4 ℃ before analysis.

3.4. Sample preparation for LC-Q-TOF-MS

3.4.1. MSPD extraction

0.1 g of sample and 0.2 g of dispersion adsorbent were placed in the agate mortar and blended using an agate pestle until a visually homogeneous dispersed mixture was obtained. The air-dried *E. fischeriana* samples used in the present study were pulverized to powder (has been pulverized through 200 mesh sieve). The complete dispersed mixture was transferred into the column (the volumn is 5 mL, the diameter is 8 mm) with a layer of absorbent cotton on the bottom(a thin layer to make sure the sample won't leak out). After fill, a thin layer of absorbent cotton was added at the top of the sample. Then the column was eluted with 10 mL of water: ethanol (30: 70, v: v) by gravity flow. The target analytes were eluted out and collected in a 25 mL brown volumetric flask, and filtered through a 0.45 μm filter membrane before analysis. Five microliters of the sample solution was injected to the instrument and separated under the chromatographic conditions.

3.4.2.Ultrasonic extraction

Finely ground powder (1.0 g) was accurately weighed and extracted with 50 mL of 70% ethanol-water solution in ultrasonic bath (power: 400 W, frequency: 37 kHz) for 30 min in 40 ℃ and filtered. This extraction was repeated once more. The combined filtrate was evaporated to dryness. The residue was then dissolved and diluted using methanol to 50 mL volumetric flask and filtered through a 0.45 μm filter membrane before analysis.

3.4.3 Reflux extraction

1.0 g of sample and 50 mL of 70% ethanol-water solution were put into a 500 mL distilling flask. The mixture was heated at 90 ℃ and refluxed for 2 h. The extract was transferred into a 50 mL of volumetric flask and diluted to the mark with ethanol. After filtration with a 0.45 μm filter membrane before analysis.

3.5. Analytical method

LC-DAD analysis was performed on a Waters Alliance UPLC system (Waters, USA), equipped with a binary solvent delivery system and Empower™ 3 software, 2489 ultraviolet detector and 2707 automatic sampler. Separations were performed on a waters ACQUITY BEH C_{18} column (2.1 mm×100 mm, 1.8 μm) operating at 30 ℃. Different mobile phase components, for example acetonitrile, methanol and aqueous were evaluated. The proportions of organic and aqueous components of the mobile phase, the pH and the flow rate were systematically varied to optimize the method. The mobile phase eventually selected was a gradient prepared from acetonitrile (component A) and 0.1% (*v*/*v*) formic acid in water (component B). The UPLC elution condition was optimized as follows: 4% A (0-5 min), 4%-35% A (5-10 min), 35%-70% A (10-25 min), 70%-80% A (25-35 min), 80%-100% A (35-40 min). The original composition was then used for 5 min to restore the initial conditions. The flow rate was set at 0.8 mL/min and the injection volume of reference compounds and samples was 5 μL. The analytes were monitored at 210 nm.

Identification of compounds in *Euphorbia fischeriana* by UPLC/Q-TOF-MS was performed with a Waters (USA) Xevo Q-TOF-MS equipped with an electrospray ionization (ESI) source. The electrospray source included one nebulizer used for the LC eluent and a second used for the internal reference solution, which consisted of solution formate introduced into the TOF-MS by means of an automated calibrant delivery system in order to obtain accurate mass measurement. Post-column sample introduction was achieved by use of a split value. UPLC/Q-TOF-MS analysis was performed in positive (ESI^+) ion mode under the operating condition: capillary voltage, 10 kV; cone voltage, 15 V; the flow rate of nebulizer gas and cone gas: 800 L/h and 50 L/h; gas temperature, 230 ℃. Full scan spectra were acquired in the mass range of m/z 50-1000. The accurate mass and molecular formula assignments were obtained with the MassLynx 4.1 software (Waters MS Technologies).

3.6. Method validation for quantification

Among the 25 identified compounds, 5 compounds were quantified by UPLC/Q-TOF-MS. Peak area was integrated at the expected retention times under full scan MS conditions.

Calibration curves (seven points) were obtained using external standard calibrations for 5 analytes injecting the mixed standard solution in the wide concentration range (A: 0.625-50 μg/mL; B: 2.5-200 μg/mL; C: 1.25-100 μg/mL; D: 2.5-200 μg/mL; E: 0.625-50 μg/mL). Calibration curves were established by plotting the peak area versus the concentration of each analyte.

The limits of detection (LODs) were estimated from the injection of a standard solution, successively diluted until reaching a concentration level corresponding to a signal-to-noise (S/N) ratio of 3. The limits of quantification (LOQs) were defined and determined as the minimum quantified amount of the analytes at a S/N ratio of about 10.

Precision of the method was checked for intraday and interday variability. The intraday variability study was carried out by the injection of the middle concentration standard solution six consecutive times in the same day. The interday variability study was carried out for three successive days using the same solution. The stability was tested with the sample at room temperature and analyzed at 0, 6, 12, 24 and 48 h within 2 days. To confirm the repeatability, six different samples solutions prepared from the same sample were analyzed. Variations were expressed by RSD.

The accuracy of the analytical method was determined by spiking into the *Euphorbia fischeriana* powder with different amounts of authentic standards with known contents of the five analytes. Then, the samples were treated according to the sample extraction procedure. Three replicates were performed for the test.

4. Discussion

This is the first time to determine the five active compounds in *Euphorbia fischeriana* using MSPD method to extract to the best of our knowledge. Comparing with traditional ways, such as Ultrasonic extraction and Reflux extraction ways, MSPD method has the advantages of time saving, less solvent consumption and no emulsification, etc. The importance, MSPD will improve the

recovery and enhance the detection capability of analytes through greatly enhancing the ability of separation and enrichment the target compounds. From the results obtained in this paper, we knew that the extraction yields of the two phenolics and three terpenoids obtained by the MSPD were higer than those of traditional reflux and sonication extraction methods. Identification of the traditional Chinese medicine is important to control the quality of the herbs and differentiate positive or negative herbs and to ensure efficacy and safely use in clinic. UPLC/Q-TOF-MS as a more and more important method in the study of traditional Chinese medicine combines high resolution, high selectivity and high separation advantages. With the development of traditional Chinese medicine, studies on the pharmacodynamic basic substances have become the focus of the whole academic community. So the MSPD and UPLC/Q-TOF-MS techniques were introduced to analyze the material basis of *Euphorbia fischeriana.* This analysis method may facilitate the scientific extraction and quality control, even facilitate the elucidation of the action mechanism of traditional Chinese medicine.

Compounds of 2,4-dihydroxy-6-methoxy-3-methylacetophenone and Jolkinolide B were also listed as the quality standard of *Euphorbia fischeriana* in the "Common and important standard for drug safety (2006BAI14B01)" [22]. Although it was contained in 2015 version Chinese Pharmacopoeia, the detection methods are obsolete and insensitive, there is even no determination items of active compounds. At the same time, it is necessary to develop a method for chemical profiling to supplement the quality control of *Euphorbia Fischeriana*. The research on effective substance basis is momentous for the modern study of the Chinese herbs. To elute the target compounds completely with the minimum volume of elution solvent, 8 mL, 10 mL and 12 mL were studied. Finally, 10 mL was chosen considering the extraction efficiency and solvent consumption.

Among the four columns (Dikma-C_{18}, Agilent-C_{18}, Waters-C_{18} column and Phenomenex-C_{18}) that were tested for the separation of the sample, Waters ACQUITY BEH C_{18} column gave the best chromatographic resolution. Among the identified compounds, the compounds 1, 2, 3 and 17 listed in table 2 can not be identified through the exact mass data according to the literatures. ~~So our next step will be preparing the compounds using semi-preparative UPLC instrument and identifying them using ^{13}C-NMR and ^{13}H-NMR methods.~~

5. Conclusions

This study has demonstrated a new method for identifying and quantifying the active compounds in *Euphorbia fischeriana.* This method combines MSPD with UPLC/Q-TOF-MS to obtain the chemical profiling, which is preferable to the QC of *Euphorbia fischeriana*. Because the clinical efficacy of traditional Chinese medicine depends on the integrated effects of the multiple components, so quantification of one or several active components in does not demonstrate its chemical natures.

The method offers advantages of shorter analytical time, less reagents consumption, and simplicity over existing systems as well as excellent selectivity and sensitivity were shown. This valuable information concerning the components and amounts of these pharmacologically active constituents in *Euphorbia fischeriana* could be of great importance for quality assessment, and should therefore be useful for the guidance of clinical use. The MSPD-UPLC/Q-TOF-MS method built in this paper could be well suited to meet quality control requirements of medicinal plants using comprehensive biochemical profiling of bioactive compounds.

Acknowledgments： The authors greatly appreciate the financial support from Heilongjiang Province University Youth Innovative Talents Training Program (Grant No: 7UNPYSCT- 201611), the National Natural Science Foundation of China (Grant No: 81403173), and the grant research project of Chinese Postdoctoral Science Foundation (Grant No: 2014M551291).

Author Contributions: W.L. and B.H. designed the research; W.L., Y.L. and Y.W. performed the research; Y.L. and B.H. analyzed the data; Y.W. and B.H. wrote the paper.

Conflicts of Interest: The authors have declared no conflict of interest.

References

1. Barrero, R.A.; Chapman, B.; Yang, Y.; Moolhuijzen, P.; Gagnère, G.K.; Zhang, N.; Tang, Q.; Bellgard, M.I.; Qiu, D.Y. De novo assembly of Euphorbia fischeriana root transcriptome identifies prostratin pathway related genes. *BMC Genomics.* **2011**, *12*, 600-606.
2. Wang, F.D.; Wan, R.D.; Yang, J.Y.; Wang, H.Q. Studies on the chemical constituents of Crassula argentea Thunb. *J. Chin. Med. Mat.* **2006**, *29*, 1184-1185.
3. Sun, Y.X.; Liu, J.C. Chemical constituents and biological activities of Euphorbia fischeriana Steud. *Chem. & Biodiv.* **2011**, *8*, 1205-1209.
4. Yang, K.; Wang, Y.S.; Lin, S.X.; Tong, D.Y. Research on the optimize treating mechanism of Langdu extract for Lewis lung cancer. *Chin. J. Can. Pre. Trea.* **2012**, *19*, 1372-1376.
5. Li, X.Q.; Chen, S.S.; Liu, S.L.; Li, M.H. Comparison of the inhibition to mycobacterium tuberculosis of different Euphorbia fischeriana extracts. *Pharm. J. Chin. Peo. Lib. Army.* **2006**, *22*, 153-155.
6. Liu, X.C.; Zhou, L.G.; Liu, Z.L. Evaluation of nematicidal activity of ethanol extracts of Euphorbiaceae plants and constituents from Euphorbia fischeriana to Meloidogyne incognita (Kofoid and White) Chitwood. *J. Ento. Zoo. Stu.* **2014**, *2*, 311-317.
7. Wang, H.B.; Chen, W.; Zhang, Y.Y.; Wang, X.Y.; Liu, L.P.; Tong, L.J.; Chen, Y. Four new diterpenoids from the roots of Euphorbia fischeriana. *Fitoterapia.* **2013**, *91*, 211-217.
8. Zhou, T.X.; Bao, G.H.; Ma, Q.G.; Qin, G.W.; Che, C.T.; Lv, Y.; Wang, C.; Zheng, Q.T. Langduin C, a novel dimeric diterpenoid from the roots of Euphorbia fischeriana. *Tetra. Lett.* **2003**, *44*, 135-137.
9. Liu, W.Z.; He, F.L.; Ran, Z.Y.; Gu, X.F.; Wu, X.Y.; Qin, G.W. Studies on chemical constituents from Euphorbia fischeriana Steud. *J. Chin. Mat. Med.* **2011**, *26*, 180-188.
10. Wang, X.Y.; Liu, L.P.; Kang, T.G.; Wang, H.B. Chemical constituents of Euphorbia fischeriana. *Chin. J. Nat. Med.* **2012**, *10*, 299-302.
11. Liang, X.; Liu, Z.G.; Cao, Y.F.; Meng, D.L.; Hua, H.M. Chemotaxonomic and chemical studies on two plants from genus of Euphorbia: Euphorbia fischeriana and Euphorbia ebracteolata. *Biochem. Syst. Eco.* **2014**, *57*, 345-349.
12. Liu, W.K.; Ho, J.C.K.; Qin, G.W.; Che, C.T. Jolkinolide B induces neuroendocrine differentiation of human prostate LNCaP cancer cell line. *Biochem. Pharm.* **2002**, *63*, 951-958.
13. Wang, X.L.; Zhou, L.; Liu, J.C. The effect of 17-hydroxyjolkinolide B on proliferation and apoptosis of K562 cells. *Chin. J. Exp. Tradit. Med.* **2013**, *19*, 197-200.
14. Xu, S.D. The antituberculosis effect of Langdu. *Zhejiang J. Integr. Tradit. Chin. West Med.* **2012**, *22*, 67-69.
15. Tian, R.J. Inhibitory effect of leaf extract of Stellera chamaejasme on skin trichophyton mentagrophytes and staphylococcus aureus. *Herald. Med.* **2014**, *33*, 729-732.
16. Manuele, M.G.; Ferraro, G.; Arcos, M.L.B.; López, P.; Cremaschi, G.; Anesini, C. Comparative immunomodulatory effect of scopoletin on tumoral and normal lymphocytes. *Life Sci.* **2006**, *79*, 2043-2048.
17. Zhang, H.Q.; Ding, Y.M. Studies on the active compounds B and C in the root of Euphorbia ebracteolata Hayata; *J. Plant Res. Envir.* **1992**, *1*, 6-9.
18. Wang, Y.; Ma, X.; Yan, S.; Shen, S.; Zhu, H.; Gu, Y.; Wang, H.; Qin, G.; Yu, Q. 17-hydroxy-jolkinolide B inhibits signal transducers and activators of transcription 3 signaling by covalently cross-linking Janus kinases and induces apoptosis of human cancer cells. *Can. Res.* **2005**, *69*, 7302-7310.
19. Luo, H.; Wang, A. Induction of apoptosis in K562 cells by jolkinolide B. *Can. J. Physiol. Pharm.* **2006**, *84*, 959-965.
20. Wang, J.H.; Zhang, K.; Niu, H.Y.; Shu, L.H.; Yue, D.M.; Li, D.; He, P. Jolkinolide B from Euphorbia fischeriana Steud induces in human leukemic cells apoptosis via JAK2/STAT3 pathways. *Inter. J. Clin. Pharm. Thera.* **2013**, *51*, 170-178.
21. Liu, G.F.; Fu, Y.Q.; Yang, Z.Q.; Zhao, H.Q.; Fan, X.M. Isolation and identification of antitumor constituents of diterpenoids lactone in Euphorbia fischeriana Steud. *Chin. Aca. J.* **1988**, *13*, 35-36.
22. Wang, C.J.; Jiang, Y.Q.; Yan, X.H. Determination of four ent-abietane jolkinolides diterpene lactones from Euphorbia fischeriana Steud. by RP-HPLC. *Chin. Tradit. Pate. Med.* **2013**, *10*, 2196-2199.
23. Wang, C.J.; Jiang, Y.Q.; Bi, F.J.; Lin, T.; Yan, X.H.; Liu, D.H. RP-HPLC determination of 2,4-dihydroxy-6-methoxy-3-methylacetophenone and jolkinolide B in Euphorbiae Ebracteolatae Radix. *Chin. J. Pharm. Anal.* **2011**, *31*, 839-842.
24. Su, X.L.; Lin, R.C.; Wong, S.K.; Tsui, S.K.; Kwan, S.Y. Identification and characterisation of the Chinese

herb Langdu by LC-MS/MS analysis. *Phytochem. Anal.* **2003**, *14*, 40-47.
25. Tang, Y.P.; Jiang, W.; Wu, Q.C.; Yu, L.; Zhang, L.; Tao, W.W.; Ding, A.W.; You, F.Q.; Duan, J.A. Comparative characteristic of the inflammatory diterpenes in the roots of Euphorbia fischeriana with different preparation method using HPLC-ELSD. *Fitoterapia.* **2012**, *83*, 427-433.
26. Barker, S.A.; Long, A.R.; Short, C.R. Isolation of drug residues from tissues by solid phase dispersion. *J. Chromatogr. A.* **1989**, *475*, 353-361.
27. Zhang, Q.; Zhu, W.Q.; Guan, H.; Liu, H.; Yang, W.Q.; Wang, H.R.; Cai, D.F. Development of a matrix solid-phase dispersion extraction combined with high-performance liquid chromatography for determination of five lignans from the Schisandra chinensis. *J Chromatogr. B.* **2016**, *1011*, 151-157.
28. Wang, Z.B.; Sun, R.; Wang, Y.P.; Li, N.; Lei, L.; Yang, X.; Yu, A.M.; Qiu, F.P.; Zhang, H.Q. Determination of phenolic acids and flavonoids in raw propolisbysilica-supported ionic liquid-based matrix solid phase dispersion extraction high performance liquid chromatography- diodearray detection Zhibing. *J.Chromatogr. B.* **2014**, *969*, 205-212.
29. Pan, L.L. The study of chemical constituents and bioactivity of Euphorbia fischeriana Steud. *Jilin Agri. Univ.* **2011**, *1*, 15-19.
30. Pan, L.L.; Fang, P.L.; Zhang, X.J.; Ni, W.; Li, L.; Yang, L.M.; Chen, C.X.; Zheng, Y.T.; Li, C.T.; Hao, X.J.; Liu, H.Y. Tigliane-type diterpenoid glycosides from Euphorbia fischeriana. *J. Nat. Prod.* **2011**, *74*, 1508-1512.
31. Zhang, G.Z.; Wang, Y.W.; Xu, H.H.; Wu, G.Q.; Zhao, S.H. Isolation and identification of extraction of Stellera chamaejasme (I). *J. Anhui Agri. Univ.* **2000**, *27*, 340-344.
32. Wang, H.; Zhang, X.F.; Ma, Y.B.; Cai, X.H.; Wu, D.G.; Luo, X.D. Diterpenoids from Euphorbia wallichii. *Chin. Tradit Herb. Drugs.* **2004**, *35*, 611-614.
33. Zou, H.Y.; Tu, P.F. Study on the chemical constituents of Lysimachia clethroides. *Chin. Tradit. Herb. Drugs.* **2009**, *40*, 704-708.
34. Su, X.F.; Lin, Q.; Huang, X.S.; Yang, J.X. Chemical constituents from the stem of Cleidiocarpon cavaleriei. *Acta Bota. Bore. Occid. Sin.* **2008**, *28*, 2339-2342.
35. Wang, W.X.; Ding, X.B. Acetophenone derivatives from Euphorbia ebracteolata. *Acta Pharm. Sin.* **1999**, *34*, 514-517.
36. Wang, Y.B.; Huang, R.; Wang, H.B.; Jin, H.Z.; Lou, L.G.; Qin, G.W. Diterpenoids from the roots of Euphorbia fischeriana. *J. Nat. Prod.* **2006**, *69*, 967-973.
37. Liu, G.F.; Lou, Z.G. Studies on chemical constituents from Euphorbia fischeriana. *Chin. Tradit. Herb. Drugs.* **1989**, *20*, 290-293.
38. Liu, X.Q.; Jia, Z.J.; Liu, Z.M. Studies on the components of Sanguisorba officinalic L. *Chem. J. Chin. Univ.* **1992**, *13*, 767-769.
39. Vitchu, L.; Kovit, C.; Kanchana, S.; Pichaet, W. Chemical constituents of Dianella ensifolia redoute. *J. Sci. Soc. Thailand.* **1982**, *8*, 95-102.
40. Wu, Q.C.; Tang, Y.P.; Ding, A.W.; You, F.Q.; Duan, J.A. Diterpenes and triterpenes from the roots of Euphorbia fischeriana. *Chin. J. Nat. Med.* **2010**, *8*, 101-102.
41. Wang, W.X.; Ding, X.B. Studies on diterpenoids from the roots of Euphorbia ebracteolata. *Acta Pharm. Sini.* **1998**, *33*, 128-131.
42. Du, Z.Z.; He, H.P.; Wu, B.; Shen, Y.M.; Hao, X.J. Chemical Constituents from the Pericarp of Trewia nudiflora. *Hel. Chim. Acta.* **2004**, *87*, 758-763.
43. Pan, Q.; Shi, M.F.; Min, Z.D. Studies on the 2D NMR spectra of Jolkinolide diterpenoids from Euphorbia fischeriana. *J. Chin. Pharm. Univ.* **2004**, *35*, 16-19.
44. Zhang, Y.Y. Studies on chemical constituents from roots of Euphorbia pekinensis Rupr. *Jilin Univ. Mast. Degr. The.* **2010**, *9*, 9.
45. Yan, X.H.; Wang, C.J.; Bi, F.J.; Gu, L.H.; Jiang, Y.Q. Improvement of quality standard of Radix Euphorbia Fischeriana. *Tradit. Chin. Drug Res. Clin. Pharm.* **2012**, *23*, 321-324.
